# An Inactivated Novel Trivalent Vaccine Provides Complete Protection against FAdV-4 Causing Hepatitis-Hydropericardium Syndrome and FAdV-8b/-11 Causing Inclusion Body Hepatitis

**DOI:** 10.1155/2023/5122382

**Published:** 2023-02-22

**Authors:** Congcong Song, Shiyi Zhao, Mingzhen Song, Qilong Qiao, Panpan Yang, Baiyu Wang, Yanfang Cong, Yanling Wang, Hongying Liu, Zeng Wang, Xinwei Wang, Jun Zhao

**Affiliations:** ^1^College of Veterinary Medicine, Henan Agricultural University, Zhengzhou 450046, China; ^2^National Animal Health Products for Engineering Technology Research Center, Qingdao 266111, China

## Abstract

Outbreaks of hepatitis-hydropericardium syndrome (HHS) caused by fowl adenovirus serotype 4 (FAdV-4) and inclusion body hepatitis (IBH) related to FAdV-8b and FAdV-11 have been increased in chickens in China since 2015. Clinical concurrent infections of FAdV-4, FAdV-8b, and FAdV-11 are quite common, yet there are no commercially available trivalent vaccines against infection by these three serotypes. In our previous study, a bivalent vaccine based on a recombinant FAdV-4, of which *fiber*-1 was replaced with the *fiber* of FAdV-8b, has been developed. In this study, a novel recombinant rFAdV-4-fiber/8b + 11 was constructed by inserting FAdV-11 *fiber* gene into the 1966-bp deletion region of rFAdV-4-fiber/8b genome. The *in vitro* replication ability of the rFAdV-4-fiber/8b + 11 was similar to the parental FAdV-4. One dose immunization with the inactivated rFAdV-4-fiber/8b + 11 vaccine generated robust immune responses against FAdV-4, FAdV-8b, and FAdV-11, and provided efficient clinical protection against FAdV-4, FAdV-8b, and FAdV-11 challenge. This study provides a novel strategy for developing potential trivalent vaccines for the prevention and control of HHS and IBH.

## 1. Introduction

Fowl adenoviruses (FAdV) belong to the genus *Aviadenovirus* of the family *Adenoviridae* [1]. FAdV have spread worldwide and are ubiquitous in poultry farms. FAdV are separated into five species (A to E) and subdivided into twelve serotypes (FAdV-1 to -8a and FAdV-8b to -11) [2] Although most of FAdV infections are subclinical, some FAdV acute infections cause clinical diseases such as inclusion body hepatitis (IBH) and hepatitis-hydropericardium syndrome (HHS) and gizzard erosions (GE) [3]. IBH mainly occurs in 3 to 4-week-old chickens with mortality approaching 10%–30% after 3-4 days [4, 5]. Since the first report of IBH in 1963, the disease has spread worldwide [6]. HHS primarily affects broilers between 3 and 6 weeks of age, causing 20%–80% mortality [7, 8]. Since the first report in Pakistan in 1987, HHS has spread to Asia, Europe, South, and North America. Epidemiological data have confirmed HHS is mainly caused by FAdV-, whereas IBH is associated with FAdV-2, -8a, -8b, and -11 [9–13].

The capsid of FAdV consists of Hexon capsomers, Penton bases, and Fiber proteins. Serotype-specific determinants are located on both the Fiber and the Hexon. FAdV-1, -4, and -10 possess two *fiber* genes with differences in length, namely *fiber*-1 and *fiber*-2, whereas other serotypes including FAdV-8a, -8b, and -11 have only one *fiber* gene in their genomes [14, 15]. Accumulating data has demonstrated that Fiber plays principal roles in initiating FAdV infection, tissue tropism, virulence, and inducing type-specific neutralization [16–19]. Fiber has been repeatedly confirmed as an effective immunogen for developing protective vaccines against different FAdV-induced diseases [20–25].

Since 2015, the outbreaks of HHS and IBH have been spreading widely in China [13, 26]. Epidemiological surveys of FAdV infections in China from 2007 to 2021 indicated that FAdV-4, FAdV-8b, and FAdV-11 were the predominant serotypes [13, 26]. Although several potential vaccines against FAdV-4, FAdV-8b, FAdV-4, and FAdV-8b, or FAdV-8b and FAdV-11, have been developed [20–24, 27–30]; no trivalent vaccine for preventing infection of FAdV-4, FAdV-8b, and FAdV-11 simultaneously has been reported so far. In our previous study, a bivalent vaccine against FAdV-4 and FAdV-8b based on a recombinant rFAdV-4-fiber/8b generated by substituting FAdV-4 fiber-1 with FAdV-8b fiber, has been developed [29]. In this study, a novel recombinant rFAdV-4-fiber/8b + 11was constructed by inserting FAdV-11* fiber* gene into the 1966-bp deletion region of the rFAdV-4-fiber/8b genome. The immunogenicity and efficacy of a potential trivalent vaccine based on rFAdV-4-fiber/8b + 11 were investigated.

## 2. Materials and Methods

### 2.1. Viruses, Cells, Plasmids, and Antibodies

The virulent FAdV-4 strain CH/HNJZ/2015 (GenBank No. KU558760), FAdV-8b strain SW2021, and FAdV-11 strain FJSW/2021 (GenBank No. OK336458) were isolated by our group and propagated in leghorn male hepatocellular (LMH) cells (ATCC, CRL-2117). LMH cells were maintained in Dulbecco's Modified Eagle's Medium/F-12 (Thermo Fisher Scientific, MA, USA) containing 10% fetal bovine serum (FBS, AusgeneX, Australia) in a 5% CO_2_ incubator at 37°C. The recombinant plasmid p15A-cm-HNJZ-fiber/8b containing the full-length genome of FAdV-4 Chinese isolate CH/HNJZ/2015 in which the FAdV-4* fiber*-1 gene has been replaced by FAdV-8b* fiber*, was constructed previously [29]. The plasmid pR6K-amp-ccdB, *E coli.* strain GBred-gyrA462, and GB05-dir were kindly provided by Professor Hailong Wang in Shandong University [31, 32]. Polyclonal rabbit sera against Fiber-2 protein of FAdV-4, Fiber protein of FAdV-8b or FAdV-11 were generated by immunizing rabbits with *E. coli*-expressed FAdV-4 Fiber-2, FAdV-8b, and FAdV-11 Fiber proteins, respectively. HRP conjugated goat antirabbit IgG is from Abcam, Cambridge, UK.

### 2.2. Construction of FAdV-4 Infectious Clone Containing the Fiber Genes of Both FAdV-8b and FAdV-11

A FAdV-4 infectious clone containing the *fiber* genes of both FAdV-8b and FAdV-11 was generated according to a protocol in our lab [19]. Briefly, the *amp-ccdB* cassette was amplified using primers 1966-ac-F and 1966-ac-R and pR6K-amp-ccdB as a template, and the FAdV-11 *fiber* gene was amplified from FAdV-11 strain FJSW2021 using primers 1966-fiber/11-F and 1966-fiber/11-R listed in Table 1. The *amp-ccdB* fragment was first cloned into the 1966-bp deletion region of the p15A-cm-HNJZ-fiber/8b to get the p15A-cm-HNJZ-fiber/8b-amp-ccdB by using LCHR strategy. Next, the *amp-ccdB* cassette was replaced by FAdV-11b *fiber* described previously [25]. The correct p15A-cm-HNJZ-fiber/8b + 11 plasmid was identified by restriction enzyme digestion and sequencing.

### 2.3. Generation and *in Vitro* Characterization of the Recombinant rFAdV-4-Fiber/8b + 11

To rescue the recombinant simultaneously expressing the Fibers of FAdV-8b and FAdV-11, LMH cells were transfected with the *Pme*I-linearized p15A-cm-HNJZ-fiber/8b + 11 using Lipofectamine 3000 (Thermo Fisher Scientific, MA, USA). The transfected cells were monitored until typical cytopathic effects appeared. The rescued chimeric virus rFAdV-4-fiber/8b + 11 was verified by PCR, sequencing, and Western blot.

### 2.4. *In vitro* Growth Properties and Stability of the rFAdV-4-Fiber/8b + 11

The rescued rFAdV-4-fiber/8b + 11 was serially passaged continuously in LMH cells. The growth kinetics of the 15^th^ passage of rFAdV-4-fiber/8b + 11 and wild-type FAdV-4 was assessed in LMH cells at a multiplicity of infection of 0.001, and the viruses were collected at 24, 48, 60, 72, 96, and 120 h postinfection (hpi), respectively. The median tissue culture infective dose (TCID_50_) of the collected viruses was determined in three technical replicates and calculated by the Reed-Muench method and presented as the mean ± standard error of mean.

The expression of FAdV-4 Fiber-2, FAdV-8b, and FAdV-11 Fiber proteins was verified by Western blotting. rFAdV-4-fiber/8b + 11, FAdV-4, FAdV-8b, and FAdV-11 infected LMH cells were harvested and lysed in RIPA lysis buffer with protease inhibitor (Thermo Fisher Scientific, MA, USA). The extracted proteins were subjected to SDS-PAGE and transferred to a nitrocellulose (NC) membrane. The NC membrane was incubated with primary antibody, and then reacted with HRP-labeled secondary antibody. After washing, the membrane was treated with ECL substrate (Millipore, Germany) and developed on Amersham Imager 600 RGB scanner.

### 2.5. Preparation of Oil-Adjuvant Inactivated rFAdV-4-Fiber/8b + 11 Vaccine

The concentrated rFAdV-4-fiber/8b + 11 (≥10^6^ TCID_50_/100 *μ*l) was inactivated with formaldehyde in 0.2% final concentration. The inactivated virus was emulsified with ADJ 501 (W/O/W) adjuvant (Zhengzhou Adjuvant Biotech CO., LTD, Zhengzhou, China) at a ratio of 1 : 1 (v/v). The final dose of rFAdV-4-fiber/8b + 11 in the oil-emulsion vaccine was 10^6^ TCID_50_ in 200 *μ*l per bird.

### 2.6. Animal Experiment

Seventy 7-day-old SPF chickens were equally divided into seven groups designated as vaccinated/challenge FAdV-4, vaccinated/challenge FAdV-8b, vaccinated/challenge FAdV-11, FAdV-4 challenge control, FAdV-8b challenge control, FAdV-11 challenge control group, and the uninfected control group.

The vaccinated chickens were inoculated intramuscularly with one dose vaccine. The control chickens were inoculated with 200 *μ*l of cell culture medium emulsified with ADJ501 adjuvant. Two weeks postimmunization, the vaccinated and challenge control chickens were challenged with either 200 *μ*l (2 × 10^5^ TCID_50_) of the virulent FAdV-4 or 200 *μ*l (2 × 10^6^ TCID_50_) of the FAdV-8b or FAdV-11, correspondingly, and the uninfected control chickens were injected with PBS. Chickens were monitored daily until 7 days postchallenge (dpc).

Serum samples were collected from all the chickens before vaccination and weekly after vaccination for evaluation of FAdV-specific antibody responses by ELISA. Cloacal swabs were collected daily postchallenge. The liver, heart, spleen, kidney, lung, cecal tonsil, pancreas, bursa of Fabricius, proventriculus, and duodenum were collected for viral load measurement. Histopathological lesions were examined in selected organs.

### 2.7. Enzyme-Linked Immunosorbent Assay (ELISA)

The antibody responses against FAdV-4, FAdV-8b, and FAdV-11 induced by the inactivated vaccine were evaluated by a FAdV-specific indirect ELISA with some modifications [33]. Briefly, ELISA plates were coated with *E. coli* expressed Fiber-2 protein of FAdV-4, Fiber protein of FAdV-8b, or FAdV-11, respectively. After washing with PBS containing 0.1% Tween-20 (PBST), diluted sera were added and incubated at 37°C for 1 h After repeated washing with PBST, the plate was incubated with rabbit antichicken IgY conjugated with horseradish peroxidase (Abcam, Cambridge, UK) at 37°C for 1 h. Then, the plate was washed again and incubated with TMB substrates (Solarbio, Beijing, China) for 10 min at 37°C. Then, 2 M sulphuric acid (Solarbio, Beijing, China) was added to each well to stop the reaction. The OD450_nm_ was measured on the Multiskan GO spectrophotometer (Thermo Fisher Scientific, MA, USA).

### 2.8. Quantification of Viral Loads and Detection of Viral Shedding

To quantify the viral loads in tissues and detect the viral shedding through cloaca, total DNA was extracted from tissue or cloacal swab samples using commercial DNA extraction kit (Tiangen, Beijing, China). The viral loads of challenged viruses were determined by a previously described SYBR Green I quantitative real-time PCR (qRT-PCR) [34]. The final viral DNA concentration was calculated as copy numbers per milligram of the tissue sample.

Viral shedding of challenge viruses was determined by conventional PCR using primers for the *fiber*-2 gene of FAdV-4, *fiber* gene of FAdV-8b, and FAdV-11, respectively (Table 1).

### 2.9. Statistical Analysis

All the data were presented as the means ± standard error of mean. Statistical analysis in this study was executed using the Unpaired *t*-test within the GraphPad 6 software. *P* < 0.05 was considered statistically significant.

## 3. Results

### 3.1. Construction and *in Vitro* Characterization of the Recombinant rFAdV-4-Fiber/8b + 11

**Figure 1:**
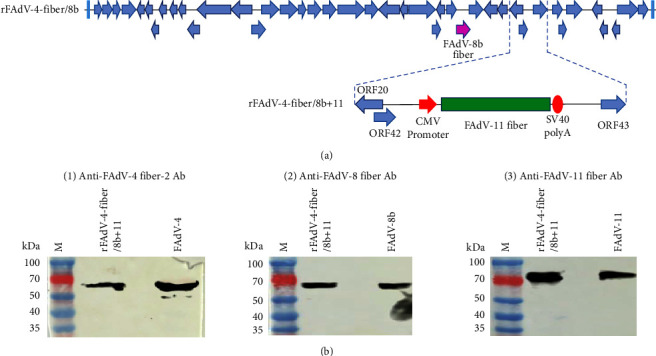
Generation of recombinant rFAdV-4-fiber/8b + 11. (a) Strategy for generation of rFAdV-4-fiber/8b + 11. (b) Identification of rFAdV-4-fiber/8b + 11 by western blot.

**Figure 2:**
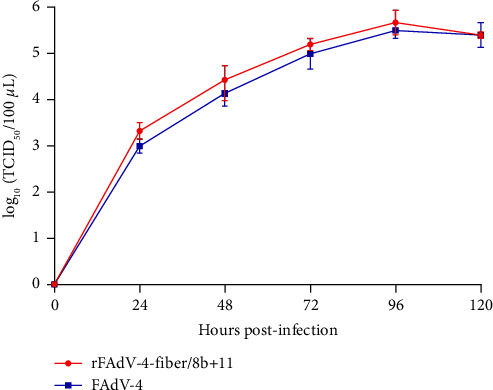
The rFAdV-4-fiber/8b + 11 replicated efficiently in LMH cells. LMH cells were infected with rFAdV-4-fiber/8b + 11 and wild-type FAdV-4 at same dose (MOI = 0.001), respectively, and the TCID_50_ of each virus collected at the indicated time points was determined in three technical replicates and presented as the mean ± standard error of mean.

To construct a recombinant FAdV-4, expressing *fiber* of both FAdV-8b and FAdV-11, a recombinant infectious clone p15A-cm-HNJZ-fiber/8b + 11 was generated by inserting the FAdV-11 *fiber* expression cassette into the 1966-bp deletion region of previously constructed infectious clone p15A-cm-HNJZ-fiber/8b as indicated in Figure 1(a). LMH cells were transfected with *Pme*I-linearized p15A-cm-HNJZ-fiber/8b + 11, and the recombinant rFAdV-4-fiber/8b + 11 was rescued successfully. The *in vitro* stability of the serially passaged rFAdV-4-fiber/8b + 11 was verified by PCR and sequencing (data not shown). The efficient expression of FAdV-4 Fiber-2 (∼53 kDa), Fibers of FAdV-8b (∼55 kDa), and FAdV-11 (∼70 kDa) by the recombinant rFAdV-4-fiber/8b + 11 was verified by Western blot in Figure 1(b). The growth kinetics of rFAdV-4-fiber/8b + 11 and wild-type FAdV-4 were determined and compared. As indicated in Figure 2, rFAdV-4-fiber/8b + 11 replicated efficiently in LMH cells with very similar replication kinetics to that of wild-type FAdV-4, and the peak titer of rFAdV-4-fiber/8b + 11 could reach to 10^5.67^ TCID_50_/100 *μ*l.

### 3.2. Inactivated rFAdV-4-Fiber/8b + 11 Vaccine Provided Efficient Cross-Protection against FAdV-4, FAdV-8b, And FAdV-11

**Figure 3:**
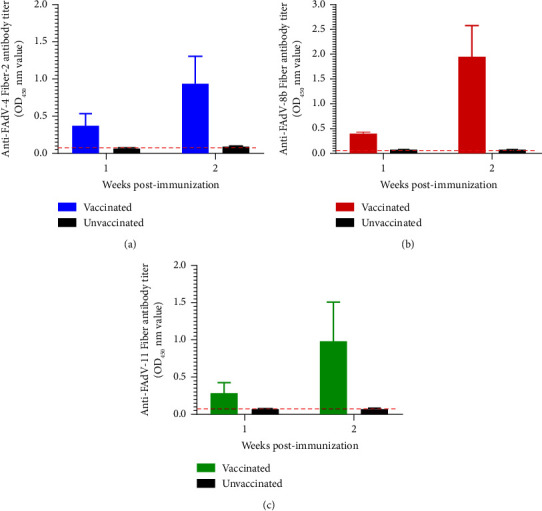
Inactivated rFAdV-4-fiber/8b + 11 vaccine induced specific immune responses against FAdV-4, FAdV-8b, and FAdV-11. The antibody responses against FAdV-4, FAdV-8b, and FAdV-11 was evaluated by an indirect ELISA. The mean OD_450_ nm absorbance values and standard error of the mean are shown for each group. The dashed line represents the background level of detection.

The efficacy of the inactivated rFAdV-4-fiber/8b + 11 as a trivalent vaccine candidate was evaluated. After immunization with one dosage of the inactivated rFAdV-4-fiber/8b + 11 oil emulsion vaccine, the antibody immune responses against FAdV-4, FAdV-8b, and FAdV-11 were assessed by FAdV-specific ELISA, respectively. As indicated in Figure 3, in the immunized groups, the antibodies against Fiber-2 of FAdV-4, Fibers of FAdV-8b, and FAdV-11 Fibers could be efficiently detected as early as week 1 and markedly increased at week 2 postimmunization (Figures 3(a)–3(c)). The relevant antibodies could not be detected in unvaccinated control chickens.

**Figure 4:**
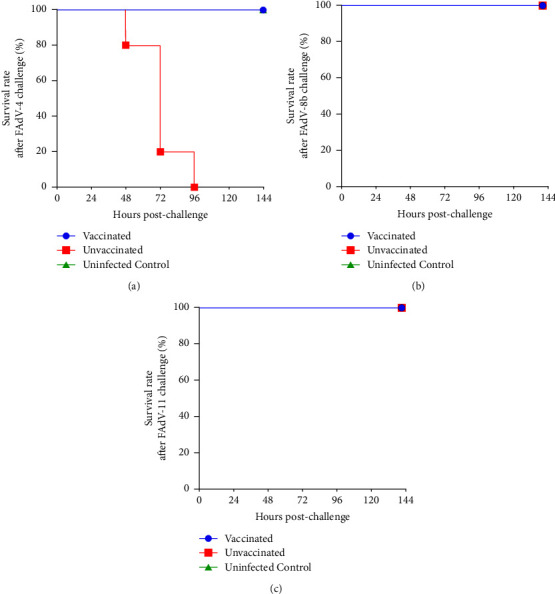
Percent of survival for chickens challenged with virulent FAdV-4 (a), FAdV-8b (b), and FAdV-11 (c). Chickens from each group were intramuscularly challenged with either 2 × 10^5^ TCID_50_ of FAdV-4 or 2 × 10^6^ TCID_50_ of FAdV-8b or FAdV-11, respectively. The challenged chickens were monitored daily for 7 days and survival rates were calculated.

Two weeks postimmunization, chickens in vaccinated/challenge and challenge control groups were challenged with either virulent FAdV-4, FAdV-8b, or FAdV-11, correspondingly. FAdV-4 challenge control chickens developed HHS-indicative clinical signs including depression and adopting a crouching position. The onset of mortality started at 48 hpc and there were no survivors at 96 hpc (Figure 4(a)). On the contrary, all vaccinated chickens survived and did not show any HHS-indicative clinical signs. Although all FAdV-8b and FAdV-11 challenged chickens survived throughout the experiment (Figures 4(b) and 4(c)), FAdV-8b or FAdV-11 challenge control chickens showed temporary loss of appetite and green watery excrement from day 2 to 5 postchallenge.

**Figure 5:**
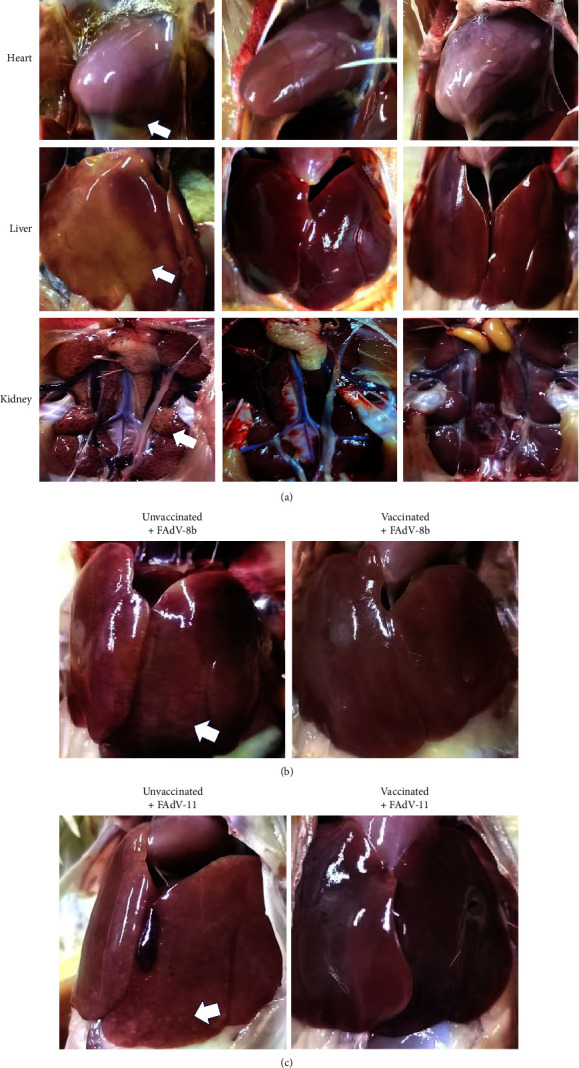
The representative gross lesion in the heart, liver, and kidney from chickens in each group after challenging with FAdV-4 (a), FAdV-8b (b), and FAdV-11 (c). (a) Chickens in the unvaccinated and FAdV-4 challenge group showed accumulation of clear straw-colored fluid in the pericardial sac, swollen and discolored liver, and enlarged kidneys with distended tubules. (b) and (c) Chickens in the FAdV-8b or FAdV-11 challenge control group exhibited IBH-indicative gross lesions, characterized by swollen livers with small white focal necrosis and petechial hemorrhages.

To further evaluated the efficacy of the trivalent vaccine, the gross and microscopic lesions, viral DNA in different tissues and viral shedding through cloaca were examined. As indicated in Figure 5(a), After FAdV-4 challenge, all sick/dead chickens in FAdV-4 challenge control group exhibited typical HHS-indicative gross lesions. In contrast, chickens immunized with the inactivated rFAdV4-fiber/8b + 11 vaccine did not show any obvious lesions (Figure 5(a)). FAdV-8b or FAdV-11 challenge control chickens groups presented typical gross IBH-indicative lesions, characterized by swollen livers with white focal necrosis or petechial hemorrhages (Figures 5(b) and 5(c)). However, no obvious gross lesions in the kidney and heart were observed in unvaccinated and FAdV-8b or FAdV-11 challenged chickens (data not shown).

**Figure 6:**
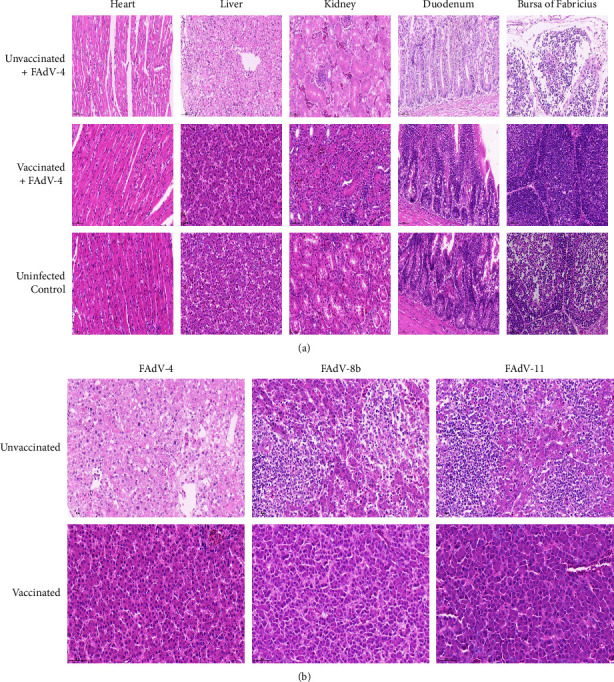
Representative histopathological lesions in tissues from chickens in each group after challenging with FAdV-4, FAdV-8b, and FAdV-11. (a) Histopathological lesions including widened myocardial cell gap and myocardial fiber rupture; severe degeneration and necrosis of hepatocytes; degeneration and necrosis of renal tubular epithelium; mucosal epithelial cell nuclear fragmentation and necrosis in duodenums; and severe depletion and necrosis of lymphocytes in the bursa of Fabricius were observed in the chickens from the unvaccinated and FAdV-4 challenge group. (b) Compared with the massive severe degeneration and necrosis of hepatocytes caused by FAdV-4, the primary histopathological lesions presented by the unvaccinated and FAdV-8b or FAdV-11 challenge groups included multifocal degeneration and necrosis of hepatocytes and mononuclear cell infiltration. However, chickens from the inactivated rFAdV4-fiber/8b + 11 vaccinated group did not present histopathological lesions of HHS and IBH mentioned above (HE staining, original magnification ×400).

The typical histopathological lesions presented by the sick/dead chickens in the FAdV-4 challenge control group included widened myocardial cell gap and myocardial fiber rupture; severe degeneration and necrosis of hepatocytes; degeneration and necrosis of renal tubular epithelia; necrosis of mucosal epithelia in duodenums; and necrosis and depletion of lymphocytes in the bursa of Fabricius (Figure 6(a)). Comparing with the massive severe degeneration and necrosis of hepatocytes caused by FAdV-4, the primary histopathological lesions presented by the unvaccinated and FAdV-8b or FAdV-11 challenge groups included multifocal degeneration and necrosis of hepatocytes and mononuclear cell infiltration (Figure 6(b)). However, the inactivated rFAdV4-fiber/8b + 11 vaccine immunized chickens did not show HHS- or IBH-indicative histopathological lesions (Figures 6(a) and 6(b)).

**Figure 7:**
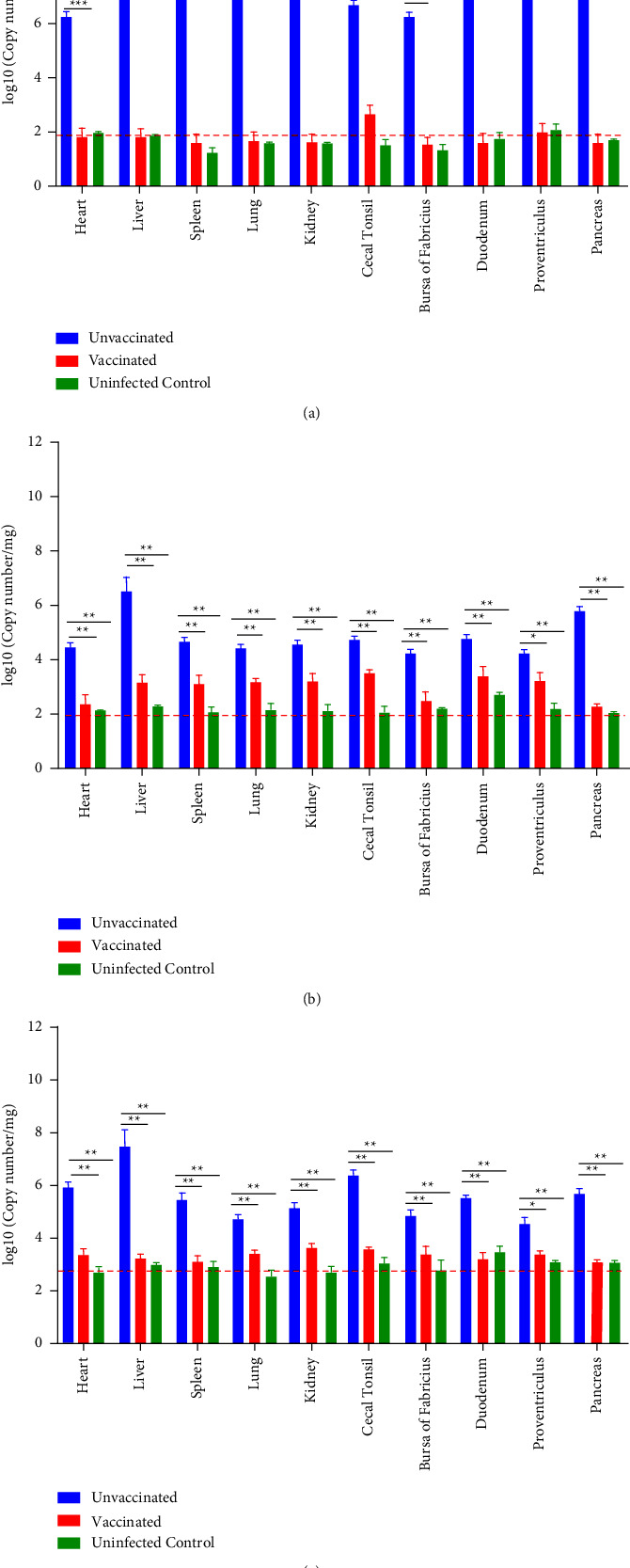
Viral loads in tissues from chickens in each group after challenging with FAdV-4 (a), FAdV-8b (b), and FAdV-11 (c). A SYBR Green qRT-PCR was used to determine the viral loads in tissues. The viral loads were calculated as copy numbers/milligram tissue and presented as the means ± standard error of the mean (^*∗*^, ^*∗∗*^, and ^*∗∗∗*^ indicate *P* < 0.05, 0.01, and 0.001, respectively). The dashed line represents the background level of detection.

As indicated in Figure 7, viral DNA copy numbers in different tissues from vaccinated chickens were significantly lower than those of challenge control chickens. Only a background level of viral loads was detected in most tissues from vaccine immunized and uninfected control chickens.

The efficacy of inactivated rFAdV4-fiber/8b + 11 vaccine protected chickens from shedding challenge virus was also assessed. As shown in Table 2, vaccinated chickens stopped excreting challenged FAdV-4, FAdV-8b, and FAdV-11 after 3, 4, and 5 dpc, respectively. All the challenge control chickens excreted the challenge viruses until the end of experiment.

## 4. Discussion

FAdV have been related with numerous disease conditions in chickens. FAdV-4 causing HHS and FAdV-8b or FAdV-11 causing IBH have spread worldwide and resulted in significant economic losses to the world poultry industry. Vaccination has been demonstrated as one of the most effective and cost-efficient infectious disease interventions that there is. However, the control of HHS and IBH turns out to be more complicated by the involvement of multiple FAdV serotypes [3]. Most HHS are caused by FAdV-4, whereas FAdV-2, FAdV-8a, FAdV-8b, and FAdV-11 are related to IBH. The outbreaks of HHS and IBH have been increased since 2015 [13, 26, 35]. There is an urgent need for developing multivalent vaccines against various FAdV serotypes causing HHS and IBH.

The Fiber protein of FAdV has been proven as an efficacious antigen for developing vaccine candidates [21, 36]. However, the intrinsic serotype-specific neutralizing activity of Fiber limits the application and efficacy of fiber-based vaccines [36]. For controlling HHS, vaccine candidates based on inactivated FAdV-4 or Fiber-2 protein have been generated and proven to be efficient [20, 22, 24, 37–42]. In order to develop potential vaccines providing broad cross-protection against HHS and IBH caused by various serotypes of FAdV, several bivalent vaccine candidates against both FAdV-4 and FAdV-8b, FAdV-8a and FAdV-8b, or FAdV-4 and FAdV-11, have been generated [23, 27–29]. De Luca et al. and Schachner et al. have developed vaccines based on chimeric fibers crecFib8b/8a or crecFib-4/11containing epitopes from fibers of FAdV-8a and FAdV-8b, Fiber-2 of FAdV-4, and Fiber of FAdV-11, respectively [23, 27]. The chimeric Fibers induced cross-neutralizing antibodies against the corresponding both serotypes and provided protection against FAdV-8a or FAdV-8b challenge and FAdV-4 or FAdV-11 challenge. These efforts provide new ideas for the development of multivalent subunit vaccines against FAdV. Lu et al. have generated a chimeric FAdV-4, FA4-F8b by inserting the *fiber* gene of FAdV-8b between *fiber*-1 and *fiber*-2 of FAdV-4, and proven the potential application of the inactivated FA4-F8b as a vaccine candidate against FAdV-4 and FAdV-8b [28].

FAdV-4 possesses two *fiber* genes that encode Fiber-1 and Fiber-2 proteins. Fiber-1 plays crucial role in mediating FAdV-4 infection and Fiber-2 plays important roles in inducing protective immune responses [37, 43, 44]. FAdV-8b and FAdV-11 only have one *fiber* gene in their genomes. Based on the above facts, we speculate that the Fiber of FAdV-8b or FAdV-11 plays dual roles in mediating viral infection and inducing effective antiviral immune responses. In our previous study, a recombinant FAdV-4, rFAdV-4-fiber/8b was successfully constructed by replacing *fiber*-1 of FAdV-4 with the *fiber* of FAdV-8b [29]. The recombinant rFAdV-4-fiber/8b maintained efficient replication capacity and pathogenicity as the parent FAdV-4. The inactivated bivalent vaccine based on the rFAdV-4-fiber/8b also provided efficient protection against FAdV-4 causing HHS and FAdV-8b causing IBH. As the 1966-bp natural deletion region and more than 20 genes in the FAdV-4 genome have been identified as potential insertion sites for foreign genes [19, 45], thus using wild type or modified FAdV-4 as a vector to express *fiber* genes from other serotypes of FAdV provides another strategy for developing multivalent vaccines for simultaneous control of HHS and IBH.

Epidemiological data have indicated that FAdV-4, FAdV-8b, and FAdV-11 are the predominant serotypes in China [25, 46, 47]. Clinical concurrent infections of various serotypes of FAdV are quite common, yet there are no trivalent vaccines for the prevention and control of HHS and IBH. In the present study, a novel recombinant rFAdV-4-fiber/8b + 11 coexpressing Fibers of FAdV-8b and FAdV-11 was constructed by inserting the *fiber* gene of FAdV-11 into the 1966-bp deletion region of the rFAdV-4-fiber/8b genome. The rFAdV-4-fiber/8b + 11 showed similar growth kinetics *in vitro* with the wild-type FAdV-4. A single immunization with the inactivated rFAdV-4-fiber/8b + 11 vaccine generated specific immune responses against FAdV-4, FAdV-8b, and FAdV-11. All immunized chickens developed full resistance to clinical disease and did not present HHS- and IBH-indicative gross and histopathological lesions. Compared with unvaccinated and challenged chickens, vaccinated chickens exhibited significantly lower viral loads in various tissues. This study reconfirmed Fiber as a critical immunogen for developing vaccines against diseases caused by various FAdV. Since both the recombinant rFAdV-4-fiber/8b in our previous study and the recombinant rFAdV-4-fiber/8b + 11 in the present study can replicate efficiently as the parent FAdV-4, it implies that the Fiber of FAdV-8b and Fiber-1 of FAdV-4 might play the similar role in initiating viral infection of target cells. In order to verify whether the Fiber of FAdV-11 may also play a similar role as Fiber-1 of FAdV-4, we are making another chimeric FAdV-4 in which *fiber*-1 of FAdV-4 will be seamlessly replaced with the *fiber* of FAdV-11.

In summary, a novel chimeric FAdV-4 coexpressing Fibers of FAdV-8b and FAdV-11 was generated for the first time. The inactivated rFAdV-4-fiber/8b + 11 could be used as a trivalent vaccine for simultaneous prevention of the concurrent infection of FAdV-4, FAdV-8b, and FAdV-11. This study provides a novel strategy for developing potential multivalent vaccines for the prevention of IBH and HHS.

## Figures and Tables

**Table 1:** Primers used for generation of recombinant virus and detection of viral shedding.

Name	Sequence (5′-3′)
1966-ac-F	AACATAAGAATCAGGGGTGGCCCGTATACTAATCCCGTCACTGACGACAC**TTAATTAA**TTTGTTTATTTTTCTAAATAC

1966-ac-R	CACTCGAGAAGGAGCCTCTGAGCCGTACTCTATGCATTGCGTGATTGTGG**TTAATTAA**TTTGTTCAAAAAAAAGCCCGC

1966-fiber/11-F	AACATAAGAATCAGGGGTGGCCCGTATACTAATCCCGTCACTGACGACACTCAATATTGGCCATTAGCCA

1966-fiber/11-R	CACTCGAGAAGGAGCCTCTGAGCCGTACTCTATGCATTGCGTGATTGTGGTACCACATTTGTAGAGGTTT

FAdV-4 fiber-2-F	TTCCACTGTTGAAGGTAGTA

FAdV-4 fiber-2-R	GTTTATCCTTTCGATTACGT

FAdV-8b fiber-F	GGAAATGACGAGCTGGACCT

FAdV-8b fiber-R	TTGGCTCCAGTTATCGGTAA

FAdV-11 fiber-F	ATGGCGAAATCGACTCCTTTC

FAdV-11 fiber-R	CAGGAGTAGAAAATGGGTCCG

^1^bold indicates *Pac*I restriction enzyme site.

**Table 2:** Viral shedding in cloacal swabs from chickens in each group.

Group	Challenge virus	Survival rate	Viral shedding (day post challenge)						
			1	2	3	4	5	6	7
Vaccinated	FAdV-4	10/10	0/10	2/10	0/10	0/10	0/10	0/10	0/10
	FAdV-8b	10/10	0/10	3/10	1/10	0/10	0/10	0/10	0/10
	FAdV-11	10/10	3/10	4/10	3/10	2/10	0/10	0/10	0/10

Unvaccinated	FAdV-4	0/10	9/10	8/8	2/2	N/A	N/A	N/A	N/A
	FAdV-8b	10/10	10/10	10/10	10/10	10/10	10/10	10/10	10/10
	FAdV-11	10/10	5/10	10/10	10/10	10/10	10/10	10/10	10/10

## Data Availability

The data used to support the findings of this study are included within the article.

## References

[1] Benkő M., Aoki K., Arnberg N. (2022). ICTV virus taxonomy profile: Adenoviridae 2022. *Journal of General Virology*.

[2] Hess M. (2000). Detection and Differentiation of Avian Adenoviruses: A Review. *Avian Pathology*.

[3] Schachner A., Matos M., Grafl B., Hess M. (2018). Fowl adenovirus-induced diseases and strategies for their control - a review on the current global situation. *Avian Pathology*.

[4] Gomis S., Goodhope A. R., Ojkic A. D., Willson P. (2006). Inclusion body hepatitis as a primary disease in broilers in Saskatchewan, Canada. *Avian Diseases*.

[5] Mo J. (2021). Historical investigation of fowl adenovirus outbreaks in South Korea from 2007 to 2021: a comprehensive review. *Viruses*.

[6] Helmboldt C. F., Frazier M. N. (1963). Avian hepatic inclusion bodies of unknown significance. *Avian Diseases*.

[7] Li P. H., Zheng P. P., Zhang T. F., Wen G. Y., Shao H. B., Luo Q. P. (2017). Fowl adenovirus serotype 4: epidemiology, pathogenesis, diagnostic detection, and vaccine strategies. *Poultry Science*.

[8] Shah M. S., Ashraf A., Khan M. I. (2017). Fowl adenovirus: history, emergence, biology and development of a vaccine against hydropericardium syndrome. *Archives of Virology*.

[9] Chandra R., Shukla S. K., Kumar M. (2000). The hydropericardium syndrome and inclusion body hepatitis in domestic fowl. *Tropical Animal Health and Production*.

[10] Hess M., Swayne D. E., Glisson J. R., McDougald L. R., Nolan L. K., Suarez D. L., Nair V. (2013). Aviadenovirus infections. *Diseases of Poultry*.

[11] Mittal D., Jindal N., Tiwari A. K., Khokhar R. S. (2014). Characterization of fowl adenoviruses associated with hydropericardium syndrome and inclusion body hepatitis in broiler chickens. *Virusdisease*.

[12] Şahindokuyucu İ., Çöven F., Kılıç H. (2020). First report of fowl aviadenovirus serotypes FAdV-8b and FAdV-11 associated with inclusion body hepatitis in commercial broiler and broiler-breeder flocks in Turkey. *Archives of Virology*.

[13] Zhao J., Zhong Q., Zhao Y., Hu Y. X., Zhang G. Z. (2015). Pathogenicity and complete genome characterization of fowl adenoviruses isolated from chickens associated with inclusion body hepatitis and hydropericardium syndrome in China. *PLoS One*.

[14] Grgić H., Krell P. J., Nagy E. (2014). Comparison of fiber gene sequences of inclusion body hepatitis (IBH) and non-IBH strains of serotype 8 and 11 fowl adenoviruses. *Virus Genes*.

[15] Griffin B. D., Nagy É. (2011). Coding potential and transcript analysis of fowl adenovirus 4: insight into upstream ORFs as common sequence features in adenoviral transcripts. *Journal of General Virology*.

[16] Schachner A., Gonzalez G., Endler L., Ito K., Hess M. (2019). Fowl adenovirus (FAdV) recombination with intertypic crossovers in genomes of FAdV-D and FAdV-E, displaying hybrid serological phenotypes. *Viruses*.

[17] Sohaimi N. M., Hair-Bejo M. (2021). A recent perspective on fiber and hexon genes proteins analyses of fowl adenovirus toward virus infectivity-A review. *Open Veterinary Journal*.

[18] Xie Q., Wang W., Li L. (2021). Domain in Fiber-2 interacted with KPNA3/4 significantly affects the replication and pathogenicity of the highly pathogenic FAdV-4. *Virulence*.

[19] Zhang Y., Liu R., Tian K. (2018). Fiber2 and hexon genes are closely associated with the virulence of the emerging and highly pathogenic fowl adenovirus 4. *Emerging Microbes & Infections*.

[20] Chen L., Yin L., Zhou Q. (2018). Immunogenicity and protective efficacy of recombinant fiber-2 protein in protecting SPF chickens against fowl adenovirus 4. *Vaccine*.

[21] Gupta A., Ahmed K. A., Ayalew L. E. (2017). Immunogenicity and protective efficacy of virus-like particles and recombinant fiber proteins in broiler-breeder vaccination against fowl adenovirus (FAdV)-8b. *Vaccine*.

[22] Ruan S., Zhao J., Yin X., He Z., Zhang G. (2018). A subunit vaccine based on fiber-2 protein provides full protection against fowl adenovirus serotype 4 and induces quicker and stronger immune responses than an inactivated oil-emulsion vaccine. *Infection, Genetics and Evolution*.

[23] Schachner A., De Luca C., Heidl S., Hess M. (2022). Recombinantly expressed chimeric fibers demonstrate discrete type-specific neutralizing epitopes in the fowl aviadenovirus E (FAdV-E) fiber, promoting the optimization of FAdV fiber subunit vaccines towards cross-protection in vivo. *Microbiology Spectrum*.

[24] Schachner A., Marek A., Jaskulska B., Bilic I., Hess M. (2014). Recombinant FAdV-4fiber-2 protein protects chickens against hepatitis-hydropericardium syndrome (HHS). *Vaccine*.

[25] Wang H., Li Z., Jia R. (2018). ExoCET: exonuclease in vitro assembly combined with RecET recombination for highly efficient direct DNA cloning from complex genomes. *Nucleic Acids Research*.

[26] Liu Y., Wan W., Gao D. (2016). Genetic characterization of novel fowl aviadenovirus 4 isolates from outbreaks of hepatitis-hydropericardium syndrome in broiler chickens in China. *Emerging Microbes & Infections*.

[27] De Luca C., Schachner A., Heidl S., Hess M. (2022). Vaccination with a fowl adenovirus chimeric fiber protein (crecFib-4/11) simultaneously protects chickens against hepatitis-hydropericardium syndrome (HHS) and inclusion body hepatitis (IBH). *Vaccine*.

[28] Lu H., Xie Q., Zhang W. (2022). A novel recombinant FAdV-4 virus with fiber of FAdV-8b provides efficient protection against both FAdV-4 and FAdV-8b. *Viruses*.

[29] Wang B. B., Song M., Song C. (2022). An inactivated novel chimeric FAdV-4 containing fiber of FAdV-8b provides full protection against hepatitis-hydropericardium syndrome and inclusion body hepatitis. *Veterinary Research*.

[30] Wang J. J., Wang S., Zou K., Zhang Y., Xu S., Yin Y. (2018). Variant serotypes of fowl adenovirus isolated from commercial poultry between 2007 and 2017 in some regions of China. *Avian Diseases*.

[31] Wang H. H., Bian X., Xia L. (2014). Improved seamless mutagenesis by recombineering using ccdB for counterselection. *Nucleic Acids Research*.

[32] Wang X., Tang Q., Chu Z. (2018). Immune protection efficacy of FAdV-4 surface proteins fiber-1, fiber-2, hexon and penton base. *Virus Research*.

[33] Tian K. Y., Guo H. F., Li N. (2020). Protection of chickens against hepatitis-hydropericardium syndrome and Newcastle disease with a recombinant Newcastle disease virus vaccine expressing the fowl adenovirus serotype 4 fiber-2 protein. *Vaccine*.

[34] Grgić H., Poljak Z., Sharif S., Nagy É. (2013). Pathogenicity and cytokine gene expression pattern of a serotype 4 fowl adenovirus isolate. *PLoS One*.

[35] Niu Y., Sun Q., Zhang G. (2018). Epidemiological investigation of outbreaks of fowl adenovirus infections in commercial chickens in China. *Transboundary and Emerging Diseases*.

[36] De Luca C., Schachner A., Mitra T., Heidl S., Liebhart D., Hess M. (2020). Fowl adenovirus (FAdV) fiber-based vaccine against inclusion body hepatitis (IBH) provides type-specific protection guided by humoral immunity and regulation of B and T cell response. *Veterinary Research*.

[37] Cao H., Hua D., Zhang H. (2022). Oral immunization of recombinant Saccharomyces cerevisiae expressing fiber-2 of fowl adenovirus serotype 4 induces protective immunity against homologous infection. *Veterinary Microbiology*.

[38] Hu J., Li G., Wang X. (2021). Development of a subunit vaccine based on fiber2 and hexon against fowl adenovirus serotype 4. *Virus Research*.

[39] Jia Z., Pan X., Zhi W. (2022). Probiotics surface-delivering Fiber2 protein of fowl adenovirus 4 stimulate protective immunity against hepatitis-hydropericardium syndrome in chickens. *Frontiers in Immunology*.

[40] Kim M. S., Lim T. H., Lee D. H. (2014). An inactivated oil-emulsion fowl Adenovirus serotype 4 vaccine provides broad cross-protection against various serotypes of fowl Adenovirus. *Vaccine*.

[41] Liu A., Zhang Y., Cui H., Wang X., Gao Y., Pan Q. (2022). Advances in vaccine development of the emerging novel genotype fowl adenovirus 4. *Frontiers in Immunology*.

[42] Zhang Y., Liu A., Cui H. (2022). An inactivated vaccine based on artificial non-pathogenic fowl adenovirus 4 protects chickens against hepatitis-hydropericardium syndrome. *Veterinary Microbiology*.

[43] Wang W. W., Liu Q., Li T. (2020). Fiber-1, not fiber-2, directly mediates the infection of the pathogenic serotype 4 fowl adenovirus via its shaft and knob domains. *Journal of Virology*.

[44] Zou X., Rong Y., Guo X. (2021). Fiber1, but not fiber2, is the essential fiber gene for. *Fowl Adenovirus*.

[45] Pan Q., Zhang Y., Liu A. (2021). Development of a novel avian vaccine vector derived from the emerging fowl adenovirus 4. *Frontiers in Microbiology*.

[46] Changjing L., Haiying L., Dongdong W. (2016). Characterization of fowl adenoviruses isolated between 2007 and 2014 in China. *Veterinary Microbiology*.

[47] Niu D., Feng J., Duan B. (2022). Epidemiological survey of avian adenovirus in China from 2015 to 2021 and the genetic variability of highly pathogenic FAdV-4 isolates. *Infection, Genetics and Evolution*.

